# *In situ* estimation of optical properties of rat and monkey brains using femtosecond time-resolved measurements

**DOI:** 10.1038/s41598-019-45736-5

**Published:** 2019-06-24

**Authors:** Yoko Hoshi, Yukari Tanikawa, Eiji Okada, Hiroshi Kawaguchi, Masahito Nemoto, Kosuke Shimizu, Tohru Kodama, Masataka Watanabe

**Affiliations:** 1 Department of Biomedical Optics, Institute for Medical Photonics Research, Preeminent Medical Photonics Education & Research Center, Hamamatsu University School of Medicine, Hamamatsu, Japan; 2 Human Informatics Research Institute, National Institute of Advanced Industrial Science and Technology (AIST), Tsukuba, Japan; 3 Department of Electronics and Electrical Engineering, Keio University, Yokohama, Japan; 4 Health Care Center, Soka University, Tokyo, Japan; 5 Department of Molecular Imaging, Institute for Medical Photonics Research, Preeminent Medical Photonics Education & Research Center, Hamamatsu University School of Medicine, Hamamatsu, Japan; 6 Sleep Disorders Project, Tokyo Metropolitan Institute of Medical Science, Tokyo, Japan

**Keywords:** Diagnostic markers, Bioenergetics

## Abstract

An accurate knowledge of tissue optical properties (absorption coefficients, *μ*_*a*_, and reduced scattering coefficients, *μ*_*s*_’) is critical for precise modeling of light propagation in biological tissue, essential for developing diagnostic and therapeutic optical techniques that utilize diffusive photons. A great number of studies have explored the optical properties of various tissue, and these values are not known in detail due to difficulties in the experimental determination and significant variations in tissue constitution. Especially, *in situ* estimates of the optical properties of brain tissue, a common measurement target in optical imaging, is a challenge because of its layer structure (where the thin gray matter covers the white matter). Here, we report an approach to *in situ* estimates of the *μ*_*a*_ and *μ*_*s*_’ of the gray and white matter in living rat and monkey brains by using femtosecond time-resolved measurements and Monte Carlo simulation. The results demonstrate that the *μ*_*a*_ of the gray matter is larger than that of the white matter, while there was no significant difference in the *μ*_*s*_’ between the gray and white matter. The optical properties of the rat brain were very similar to those of the monkey brain except for the *μ*_*a*_ of the gray matter here.

## Introduction

Recent advances in optical imaging and optogenetic modulation techniques have greatly contributed to the evolution of life sciences^1–3^. Especially, super-resolution microscopy, which enables us to obtain molecular-graded information from *in vitro* systems, including cell suspensions, culture cells and tissue slices, is expected to improve medical diagnostics and treatment strategies^4,5^. By contrast, it has long been challenging to image and modulate biological processes deep within living tissue, and optical diagnostic (e.g. diffuse optical tomography, DOT^6^) and therapeutic techniques (e.g. photodynamic therapy, PDT^7^) with near-infrared light remain under development. This is mainly attributed to the fact that multiple light scattering occurs in biological tissue, which makes it difficult to reconstruct images from boundary measurements and focus light deeper inside tissue. Developing the diagnostic and therapeutic optical techniques requires precise modeling of light propagation in biological tissue, for which accurate knowledge of tissue optical properties (the absorption, *μ*_*a*_, and reduced scattering coefficients, *μ*_*s*_*’*) is critical^8–10^. A great number of studies have reported the optical properties of various tissue, which have typically been measured *in vitro*, and these values are not known in detail due to difficulties in the experimental determination and significant variations in tissue constitution^11,12^. Optical properties of extracted tissue easily change due to non-physiological conditions, such as the absence of blood circulation, and *in situ* measurements are essential when estimating these values. Brain tissue is one of the most common measurement targets in optical imaging and *in situ* estimates of its optical properties is a challenge because of its layer structure (where the thin gray matter covers the white matter).

Various methods, such as time-resolved measurements, spatially resolved measurements and frequency resolved measurements, have been proposed to measure the optical properties of the human brain^13–16^. In general, the gray and white matter are not separately measured and average optical properties of a large volume of tissue are estimated. Unlike these conventional approaches, Bevilacqua *et al*.^17^ tried to optically differentiate a small tissue heterogeneity. To estimate the optical properties in a small volume of tissue, of the order of a few cubic millimeters, they proposed spatially resolved reflectance measurements with small source-detector separations, from 0.3 to 1.4 mm, and applied it to human brain measurements during brain surgery. Using Monte Carlo (MC) simulations, this method needs to determine parameter *γ* = (1−*g*_1_)/(1−*g*_2_), where *g*_1_ and *g*_2_ are the first and the second moments of the phase function, respectively, to estimate the *μ*_*a*_ and *μ*_*s*_’. However, systematic errors occur unless the parameter *γ* is determined with sufficient accuracy, and for typical tissues, such as brain, the average probe depth is ~1 mm.

In this study, we used time-resolved measurements as an approach to *in situ* separate measurements of the optical properties of cerebral gray and white matter. In the time-resolved measurements, the tissue is irradiated by ultrashort (femtosecond order in the system used here) laser pulses, and the intensity of the light traveling through the tissue is recorded over time with high temporal resolution. This temporal profile of the detected light intensity is called a temporal point spread function (TPSF), the temporal probability function of all detected photons. The optical properties can be determined by fitting the measured TPSF and theoretical data derived from numerical models of light propagation in biological tissue, such as the radiative transfer equation (RTE) and the photon diffusion equation (PDE), the diffusion approximation of the RTE^18,19^. For the aim of the present study, optical fibers need to be inserted into the brain. Considering the ethics of animal research, rats were used in this study, and one monkey which had been used for electrophysiological studies and was scheduled for sacrifice, was also measured for comparisons between rodents and primates.

The rat brain is very small and the cerebral cortex of rats and monkeys is very thin (rats, 1–2 mm; monkeys, 1.5–4 mm)^20,21^. As the maximum penetration depth of detected light in the reflectance mode is positively dependent on the distance between irradiating and detecting optical fibers (*ρ*)^22–24^, the *ρ* has to be the shortest possible when measuring the cerebral cortex selectively in the reflectance mode. In the present study, very fine optical fibers (diameter, *ϕ*, 125 μm) were inserted into the brain at the *ρ* of 1.25 mm with ferruled fibers. The diffusion approximation is invalid in the vicinity of the light source, which is generally positioned within about ten millimeters in the case of biological tissue, while the RTE accurately describes light propagation even in areas close to the light source^25,26^. However, because of the very heavy computational load in solving the three-dimensional RTE, we created a look-up table (LUT) with MC methods and fitted the simulated TPSF to the measured data assuming that the brain volume of interest is an optically homogeneous medium. In this study, the optical properties at a wavelength of 800 nm, which is near the isosbestic point of oxygenated and deoxygenated hemoglobin, were estimated. Prior to the measurements of rat and monkey brains, the validity of our approach was examined by measurements of a liquid phantom.

## Results and Discussion

### Phantom experiments

The validity of our approach was examined by measurements of a liquid phantom consisting of latex particles (diameter, 0.67 μm; Latex microsphere suspensions 5067A, Thermo Scientific, USA) and food color (gardenia dye green, Kyoritsu-foods, Japan), where the *μ*_*a*_, *μ*_*s*_, and *g* are 0.0231 mm^−1^, 15.6 mm^−1^, and 0.836 at 800 nm, respectively, with optical fibers that were the same as those used in the animal experiments at the same distance between irradiating and detecting optical fibers (*ρ* = 1.25 mm).

**Figure 1:**
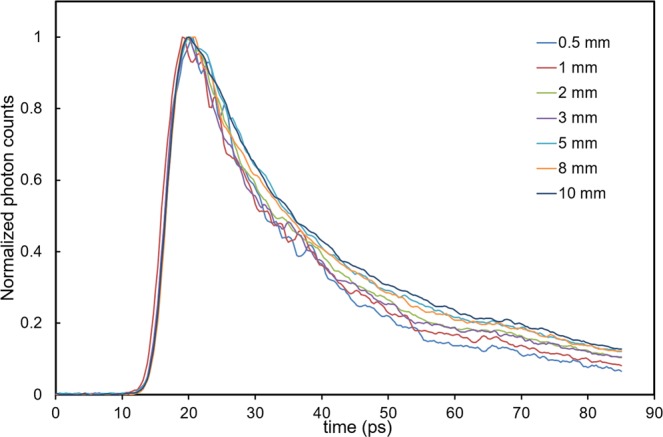
Temporal point spread functions (TPSFs) measured at different depths in the liquid phantom.

Figure 1 shows the TPSFs, which are normalized by the peak values, measured at different depths from the surface of the liquid phantom (depth of 0.5–10 mm). Although the phantom is homogeneous, the TPSFs vary with depth for reasons thought to be mainly due to the reflection at the liquid-air interface. This assumption was supported by the fact that we had initially created the LUT without considering this reflection, prior to the present study, but no simulated TPSF fit the measured ones. To get around this, we created the LUT considering the reflection at the liquid-air interface as described below (Methods, *Monte Carlo Simulation*).

**Figure 2:**
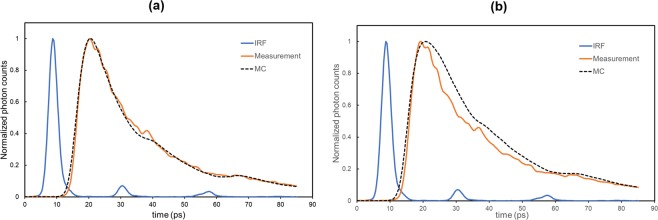
Plot of the experimental and Monte Carlo simulated temporal point spread function (TPSF) measured at two different depths in the liquid phantom. (**a**) The tips of optical fibers at the depth of 0.5 mm. Solid brown line, experimental TPSF (measurement); Dotted line, simulated TPSF (MC). (**b**) The tips of the optical fibers at the depth of 1.1 mm. The MC simulated TPSF was convoluted by the instrument response function (IRF).

Figure 2(a) shows the measured TPSF and the simulated TPSF convoluted by the instrument response function (IRF), where the full width half maximum (FWHM) was 3.44 ps, at a depth of 0.5 mm. The simulated TPSF fitted the measured data well in the range of 10% of the peak value on the leading and falling edges (10/10 fitting range, time range <85 ps), where the root mean square error (RMSE) was 0.0252. However, when the optical fibers were inserted deeper than 1 mm, the simulated TPSF did not fit the measured data (Fig. 2(b)). This can be explained by the reflection of light at the interface between the optical fibers and the liquid. Although the beams of such reflected light are weak, it is conceivable that their contribution to the detected light is not insignificant under the present experimental conditions, where the φ of the optical fibers (125 μm) is comparable to 10% of the *ρ* (1.25 mm). Considering this, the measurements were performed at the depths of 0.5 mm and 0.9 mm for the gray and white matter, respectively.

### Animal experiments

**Figure 3:**
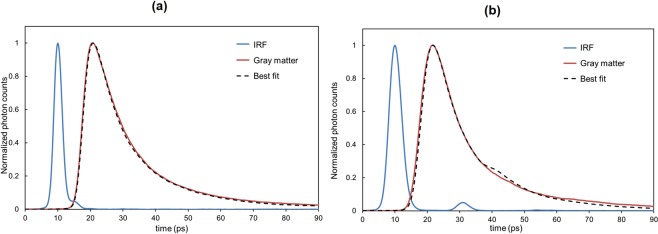
Example of the curve fitting. (**a**) The experimental TPSF (solid brown line) was obtained from measurements of the rat gray matter (primary somatosensory cortex). From the best-fit simulated TPSF (dotted line), the *μ*_*a*_ and *μ*_*s*_’ were estimated as 0.052 mm^−1^ and 1.70 mm^−1^, respectively. (**b**) The experimental TPSF (solid brown line) was obtained from measurements of the monkey gray matter (left caudal area of the inferior parietal lobule, 7a). From the best-fit simulated TPSF (dotted line), the *μ*_*a*_ and *μ*_*s*_’ were estimated as 0.081 mm^−1^ and 1.8 mm^−1^, respectively.

The simulated TPSF of the best fit was defined as the one with the minimum RMSE less than 0.025 at a 10/10 fitting range in the rats. Figure 3(a) shows the IRF, the TPSF measured at the depth of 0.5 mm of the rat brain (right primary somatosensory cortex, S1HL), and the best-fit simulated TPSF, from which the *μ*_*a*_ and *μ*_*s*_’ of the gray matter in this rat were estimated at 0.052 mm^−1^ and 1.7 mm^−1^, respectively. Three of 11 rats were excluded from the data analysis, because the insertion of optical fibers into the brain caused slight bleeding in two rats and one rat breathed spontaneously while receiving mechanical ventilation, which made the rat oxygenation difficult.

Figure 3(b) shows the IRF and the TPSF measured at the depth of 0.5 mm for the monkey brain (left caudal area of the inferior parietal lobule, 7a) and the best-fit simulated TPSF with the *μ*_*a*_ of 0.081 mm^−1^ and the *μ*_*s*_’ of 1.8 mm^−1^. In the measurements of the monkey brain, the IRF and the simulated TPSF convoluted by the IRF have a humped shape at the falling edge. The humped shape of the IRF is likely due to the reflection at the lens in the streak camera. However, the humped shape was not observed in the experimental TPSF, which can be explained by the fact that the intensity of the detected light after passing through the brain tissue was much weaker than that in the measurements of the IRF. To avoid this hump, the fitting was performed in the range of 40% of the peak value at the leading and falling edges (40/40 fitting range).

Results are detailed in Table 1, with the upper half showing the averages of the *μ*_*a*_ and *μ*_*s*_’ of the gray and white matter of the eight rats. There is significant difference in the *μ*_*a*_ value of the gray and white matter (p = 0.02): the *μ*_*a*_ value of the gray matter is larger than that of the white matter, while the mean *μ*_*s*_’ value of the gray matter is smaller than that of the white matter, though this difference is not statistically significant (P = 0.48). The larger *μ*_*a*_ of the gray matter is thought to be due to higher regional cerebral blood volumes (CBV) in the gray matter than in the white matter^27^.

**Table 1:** Optical properties of the rat and monkey brains.

Rat (n = 8)	*μ*_*a*_ (SD) [mm^−1^]	*μ*_*s*_’ (SD) [mm^−1^]
Gray matter	0.054 (0.017)	1.67 (1.09)
White matter	0.029 (0.015)	2.13 (1.11)
Monkey (n = 1, 3 brain regions)	*μ*_*a*_ (SD) [mm^−1^]	*μ*_*s*_’ (SD) [mm^−1^]
Gray matter	0.084 (0.012)	1.97 (0.29)
White matter	0.021 (0.017)	2.40 (0.60)

The lower half of Table 1 shows the averages of the *μ*_*a*_ and *μ*_*s*_’ of the gray and white matter of three different brain regions of the monkey (bilateral visual cortices, V1, left 7a, and the subcortical regions). Like the rats, the *μ*_*a*_ value of the gray matter is larger than that of the white matter (p = 0.006), while the mean *μ*_*s*_’ of the gray matter is smaller than that of the white matter, though the difference is not statistically significant (P = 0.34). In contrast to the present results, higher values of scattering coefficients (*μ*_*s*_) of the white matter than the gray matter have been reported by *in vitro* measurements of postmortem human brain tissues using an integrating sphere system^28^ and quantitative phase imaging of mouse brain tissue slices^29^. Lee *et al*.^29^ argues that this result agrees with the fact that the white matter contains more lipid than the gray matter^30^ and the lipid contents cause high local contrast of the refractive index, resulting in high values of *μ*_*s*_. No significant differences in the *μ*_*s*_’ between the gray and white matter in the present study may be attributed to low statistical power (β). However, since the standard deviation (SD) of the *μ*_*s*_’ values is large, which is consistent with the above-mentioned study with the integrating sphere system^28^, increasing the sample size to about 100 rats are required to achieve sufficient statistical power (β = 0.8).

The optical properties of the rat brain are very similar to those of the monkey brain except for the *μ*_*a*_ value of the gray matter (P = 0.02). The higher *μ*_*a*_ value of the monkey gray matter could be explained by the differences in CBV between rats and monkeys (monkey, 3.5 ml/100 g brain tissue in normocapnia^31^; rat, 2.51 ml/100 g at hematocrit of 42%^32^). Although further investigations are required, the present data suggest that the optical properties of rodent brain could be used as reference data for estimating those of human brain.

Several MC simulations^33–36^ on light propagation in the brain have used the optical properties estimated by van der Zee *et al*.^28^, who measured postmortem (40~100 hours) brain samples from three adults in the 500 to 1000 nm wavelength range with an integrating sphere. In that study, for example, the *μ*_*a*_ and *μ*_*s*_’ values of the gray matter at 780 nm were estimated at 0.036 mm^−1^ and 2.31 mm^−1^, respectively. The *μ*_*a*_ and *μ*_*s*_’ values of the white matter were 0.016 mm^−1^ and 9.25 mm^−1^, respectively. The relative differences in the optical properties between the gray and white matter show close agreement with those observed in the present study, but the large differences in the absolute values indicates the necessity of conducting *in situ* estimation of optical properties.

## Conclusions

The MC simulation on the liquid phantom described above demonstrated that light passing through a 1 mm-thick layer below the optical fiber tips makes the largest contribution to the TPSF in the time range of 0 to 85 ps, while light reaching more than 2 mm below the fiber tips contributes only little to the TPSF. According to rat^37^ and monkey brain stereotaxic atlases^38^, the measured cortical thickness was about 1.75 mm in the rats (S1HL) and about 1.8 mm (V1) and 3 mm (7a) in the monkey. From the above, it is concluded that the present approach can estimate the optical properties of the gray matter, while the estimates of the white matter are influenced by light passing through the gray matter. To estimate the optical properties of the white matter more accurately, optical fibers need to be inserted into the white matter. In the next step and based on the results here, we will develop an MC code that considers the boundary conditions between the brain and the optical fibers, creating a new LUT.

Our approach can be applied to estimate the optical properties of human thin tissues like skin as well as other rat tissues and organs. In addition, the estimation of the optical properties in a small volume of tissue has potential for clinical use, such as endoscopic optical biopsy.

## Methods

### Surgical procedures on the rats and monkey

Eleven male rats (Sprague Dawley, 10–15 weeks, 395–550 g) were anesthetized with isoflurane (4% during induction, 1.5–2.5% during surgery). Then tracheotomized, and a femoral vein and artery were cannulated to infuse drugs and examine blood gases. The rats were paralyzed with an intravenous injection of pancuronium bromide (0.02 mg/100 g body weight) and mechanically ventilated. The tidal volume and respiratory rate were adjusted to give PaCO_2_ values of 40 ± 5 mmHg when the animals were ventilated with 60% N_2_ and 40% O_2_. They were placed in a stereotaxic frame and an open-skulled cranial window was created over the left or right primary somatosensory cortex (S1HL). The brain damage caused by the optical fiber insertion was minimized (Supplementary Fig. 1).

Ketamine (10 mg/Kg) was intramuscularly administrated to a male monkey (Macaca, 11 years old) prior to the anesthesia and then the monkey was anesthetized by intravenous injection of pentobarbital (75 mg/Kg). The monkey was placed in a stereotaxic frame and breathed spontaneously. Open-skulled cranial windows had been created over the bilateral visual cortices (V1) and the left caudal area of the inferior parietal lobule (7a) for electrophysiological experiments. All the animal experiments were conducted in compliance with the protocol which was reviewed by the Institutional Animal Care and Use Committees and approved by the Presidents of Tokyo Metropolitan Institute of Medical Science and Hamamatsu University School of Medicine.

**Figure 4:**
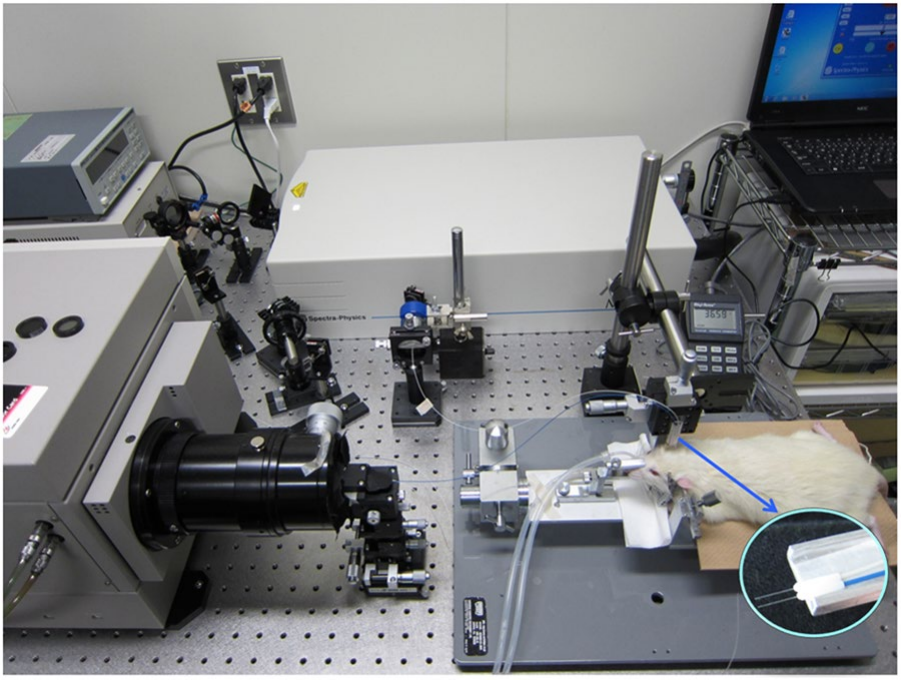
Experimental set-up. The inserted photo at the lower right shows optical fibers and ferules.

### Femtosecond Time-Resolved Spectroscopy System

A variable wavelength femtosecond laser system (Maitai, SpectraPhysics, USA) was used as the light source (Fig. 4). Laser pulses at 800 nm (FWHM < 70 fs, 80 MHz) through a bandpass filter (central wavelength, 800 nm) are divided into two paths by a beam splitter. One path is for a trigger signal to a streak camera, and the other is for measurements. The latter path is also divided into two paths: one is to an object and the other is a reference signal. Irradiating and detecting optical fibers (OM4, AISAN, Japan; core diameter, 50 μm; clad diameter, 125 μm; NA, 0.2) with a separation (*ρ*) of 1.25 mm were inserted into the brain tissue (left or right primary somatosensory cortex in the rat brain, bilateral V1 and left 7a in the monkey brain) and measured at different depths, and controlled by a micro manipulator (MMO-220A, Narishige, Japan). The *ρ* of 1.25 mm was attributed to the fact that the smallest ferrule diameter was 1.25 mm (CFLC126-10, Thorlabs, USA, inserted photo in Fig. 4). The detecting light was transferred to a streak camera (temporal resolution, 312.5 fs; C5680-12S, Hamamatsu Photonics, Japan) through the optical fiber.

The instrument response function (IRF) was measured by placing the irradiating optical fiber to be in connection with the detecting optical fiber on a black rectangular bar (40 × 100 × 20 mm, ABS resin) that has a groove in the middle for guiding and aligning the optical fibers. The tip of each optical fiber was inserted into the back opening of a ferrule and both ferrule end-faces were joined together. Then, a transparent plate (40 × 40 × 5 mm, acrylic resin) was placed on the ferrules and the four corners were fastened with screws (Supplementary Fig. 2).

We measured the brain at one depth three or more times, and an averaged curve of these TPSF was used to estimate the optical properties. It took few minutes to record one TPSF with the streak camera because of the analog data acquisition and corrections (background, shading and jitter corrections). Brain activity fluctuates even under anesthesia; however, the values reported here are averaged values over about 10 minutes.

### Monte carlo simulation

Light propagation in half-infinite homogeneous models was predicated by the MC simulation using the variance reduction technique^39^ to obtain the TPSFs for the LUT. The *μ*_s_’ and *μ*_*a*_ of the models were varied from 0.6 to 4.0 mm^−1^ with intervals of 0.1 mm^−1^ and from 0 to 0.1 mm^−1^ with intervals of 0.001 mm^−1^. The TPSFs for the models with the same scattering coefficient but different absorption coefficients were calculated from the results of the light propagation in the model without absorption (*μ*_*a*_ = 0) to reduce the computation load. The initial positions of the photon packets and incident angles were randomly changed in a 50 μm circular area and within 11.5°, respectively, to simulate the incident light from the fiber tip. The free path length of the photon propagation to the next scattering event was determined by a random number in the range [0, 1] and the scattering coefficient (*μ*_*s*_). At the scattering point, the direction of the photon propagation was recalculated by the phase function and random numbers. The phase function was calculated using the Henyey-Greenstein function with the anisotropy factor *g* = 0.9. The refractive index of the model was 1.4, and the reflection and refraction caused by the refractive index mismatch at the boundary of the model were considered. If the position and exit angle of a photon packet transmitted in air met the detection condition of the fiber, the ultimate weights of the photon for the models with different absorption coefficients were calculated by the following equation:

1 $$W\,={W}_{0}exp(-\,{\mu }_{a}\cdot L)={W}_{0}exp[-{\mu }_{a}\cdot \sum _{i=0}^{M}{l}_{i}]$$

where *W* and *W*_0_ are the ultimate weight and the weight of the transmitted photon packet, respectively, *L* is the total optical path length of the detected photon packet, *M* is the number of the scattering events, and *l*_*i*_ is free path length to the *i* th scattering event. The detecting optical fiber was placed at a distance of 1.25 mm from the incident fiber. The diameter and NA of the detecting fiber were 50 μm and 0.2, respectively. The weight of detected photon packets was accumulated into time-resolved arrays for absorption coefficients. The duration of an array element was 312.5 fs, corresponding to the temporal resolution of the streak camera. The weight of the photon packets with time-of-flight less than 300 ps was accumulated. The calculation was terminated when the results of 500,000 photon packets were accumulated in the array. The accumulated weights of the time-resolved detected photons were normalized by the maximum value in the array to obtain the TPSF for the LUT.

### Statistical analysis

The differences in the optical properties between the gray and whiter matter and between the rats and the monkey were assessed using the Welch’s t-test. P value < 0.05 was considered statistically significant. Statistical power was evaluated by using JMP (SAS Institute Inc.)

## Supplementary information

In situ estimation of optical properties of rat and monkey brains using femtosecond time-resolved measurements

## Data Availability

All data needed to evaluate the paper are available.
