# Supplementary material for: In situ estimation of optical properties of rat and monkey brains using femtosecond time-resolved measurements
Source: Sci Rep. 2019 Jun 24;9:9165. doi: 10.1038/s41598-019-45736-5 (PMC6591507; doi:10.1038/s41598-019-45736-5)
Supplement: Supplementary file 1 — In situ estimation of optical properties of rat and monkey brains using femtosecond time-resolved measurements [file 41598_2019_45736_MOESM1_ESM.pdf]

Supplementary information

In situ estimation of optical properties of rat and monkey brains using femtosecond time-resolved measurements

Yoko Hoshi, Yukari Tanikawa, Eiji Okada, Hiroshi Kawaguchi, Masahito Nemoto, Kosuke Shimizu, Tohru Kodama, Masataka Watanabe

Supplementary Figure 1

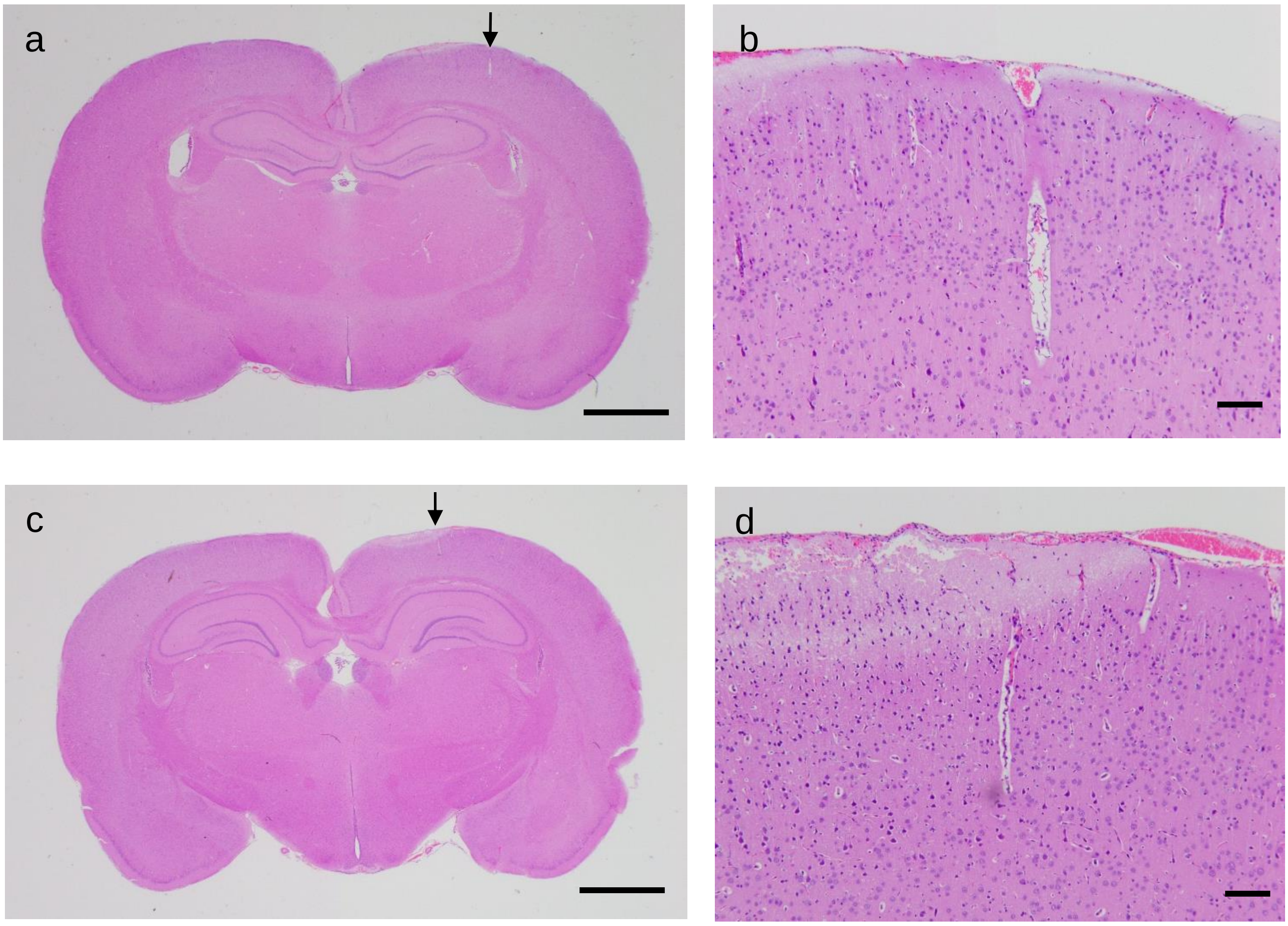

Supplementary Figure 1 Histological assessment following insertion of optical fibers into the rat brain

Two optical fibers were placed at the right somatosensory area of the rat brain in sagittal direction and inserted into the depth of 1 mm. After euthanasia, the rat brain was removed, formalin-fixed for 24 hours and sectioned to 4 μm thick slices, which were stained with hematoxylin and eosin. **a**, caudal optical fiber track (an arrow); **b**, zoomed area around the caudal optical fiber track; **c**, rostral optical fiber track (an arrow); **d**, zoomed area around the rostral optical fiber track. Bars in **a** and **c** are 2 mm. Bars in **b** and **d** are 100 μm.

Supplementary Figure 2

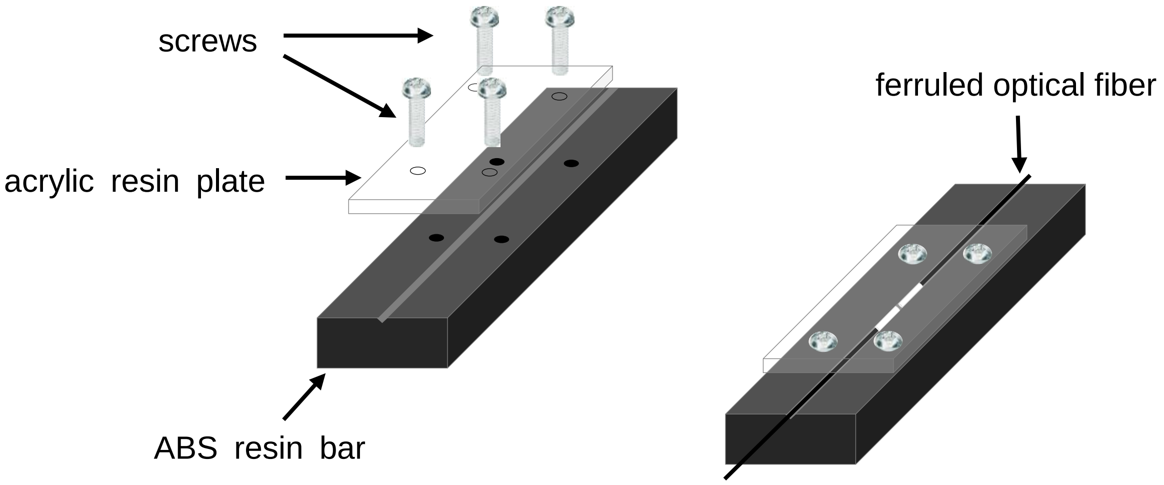

Supplementary Figure 2 Measuring the instrument response function (IRF)
